# Vibration detection: its function and recent advances in medical applications

**DOI:** 10.12688/f1000research.22649.1

**Published:** 2020-06-17

**Authors:** Tamás Oroszi, Marieke J.G. van Heuvelen, Csaba Nyakas, Eddy A. van der Zee

**Affiliations:** 1 Molecular Neurobiology, Groningen Institute for Evolutionary Life Sciences (GELIFES), University of Groningen, Groningen, The Netherlands; 2 Research Center for Molecular Exercise Science, University of Physical Education, Budapest, Hungary; 3 Department of Human Movement Sciences, University of Groningen, University Medical Center Groningen, Groningen, The Netherlands

**Keywords:** Whole body vibration, cognition, translational research, Meissner corpuscles, Pacinian corpuscles, exercise, somatosensory, brain

## Abstract

Vibrations are all around us. We can detect vibrations with sensitive skin mechanoreceptors, but our conscious awareness of the presence of vibrations is often limited. Nevertheless, vibrations play a role in our everyday life. Here, we briefly describe the function of vibration detection and how it can be used for medical applications by way of whole body vibration. Strong vibrations can be harmful, but milder vibrations can be beneficial, although to what extent and how large the clinical relevance is are still controversial. Whole body vibration can be applied via a vibrating platform, used in both animal and human research. Recent findings make clear that the mode of action is twofold: next to the rather well-known exercise (muscle) component, it also has a sensory (skin) component. Notably, the sensory (skin) component stimulating the brain has potential for several purposes including improvements in brain-related disorders. Combining these two components by selecting the optimal settings in whole body vibration has clear potential for medical applications. To realize this, the field needs more standardized and personalized protocols. It should tackle what could be considered the “Big Five” variables of whole body vibration designs: vibration amplitude, vibration frequency, method of application, session duration/frequency, and total intervention duration. Unraveling the underlying mechanisms by translational research can help to determine the optimal settings. Many systematic reviews on whole body vibration end with the conclusion that the findings are promising yet inconclusive. This is mainly because of the large variation in the “Big Five” settings between studies and incomplete reporting of methodological details hindering reproducibility. We are of the opinion that when (part of) these optimal settings are being realized, a much better estimate can be given about the true potential of whole body vibration as a medical application.

## Introduction

Vibrations are oscillations that occur around an equilibrium point. They propagate in a certain medium (such as air, water, a branch of a tree, a leaf, or soil). The waveforms of the vibrations can range from very regular (sinusoidal) to very irregular (random). Such vibrations are all around us, produced, for example, by the wind, thunder in the air, or rain drops falling on the ground. They can also be caused by organisms of any size. Vibrations are often produced unintentionally while these creatures move around. Three basic forms of vibration transmission can be distinguished based on the medium in which the vibrations propagate: airborne, water-borne, and substrate-borne vibrations. These vibrations are all intricate components of the natural environment. It is assumed that they played an important role in the evolution of life ^1^. Sensitivity to vibrations is found in even the simplest forms of life. Like the other senses, vibration detection is a crucial sense to be able to keep in touch with one’s environment ^2^. The vibration sense is used across the animal kingdom for various reasons: to detect prey, to avoid predators, to assess and navigate within a habitat or environment, or to search for food, amongst others. Vibrations are also used intentionally, for example to communicate with other individuals or to ward off a predator ^3, 4^. Also, humans detect vibrations, and even more so when other senses fail ^5^. A present-day example is feeling a vibrating mobile phone in your hand or pocket. Also, sound is a form of vibration, which is collectively referred to as vibro-acoustics. A sound (or loud noise) can be both felt and heard by humans if both hearing sense and vibration sense are present. Given our sensitivity to vibration, it is somewhat surprising that we do not employ our capacity of vibration detection to the fullest. In this paper, we highlight one approach to employ vibrational sense: the use of whole body vibration (WBV) and its potential for medical applications. We summarize the latest advances in the use of WBV and stress that WBV has great potential as a therapeutic treatment once some critical issues are solved.

## Vibration detection in humans

**Figure 1.**
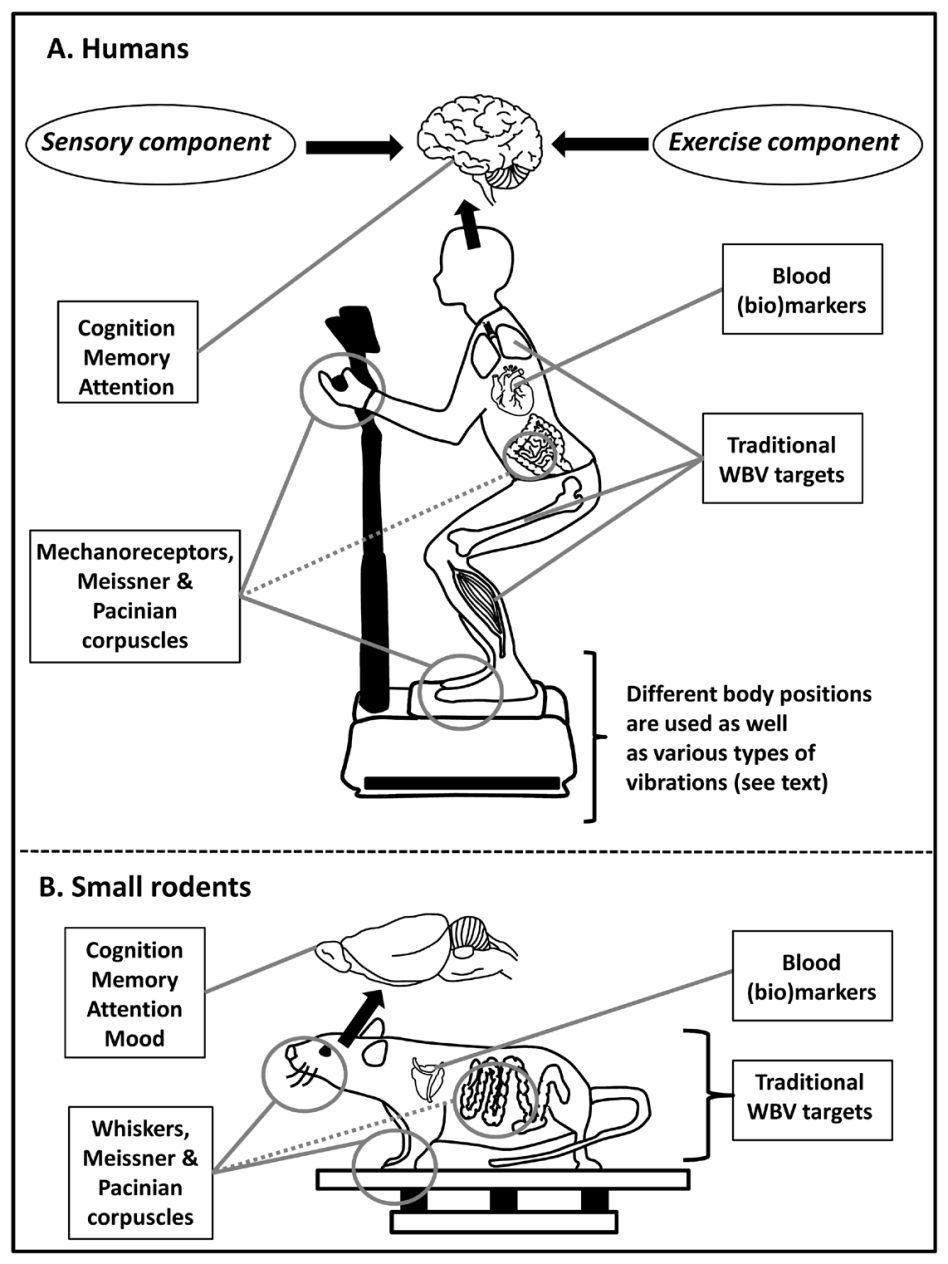
Human and translation research on whole body vibration (WBV). A schematic and simplified overview of the sensory and exercise components of WBV in humans (panel A) and small rodents (panel B) applied via a vibrating platform. The (mechanical) vibration has predominantly a sinusoidal waveform (potentially beneficial), as more random and erratic waveforms are potentially harmful (see also the European vibration directive ^14^). General focus areas in WBV research are indicated in the boxes. The dashed line from the gut microbiome indicates that WBV might directly affect the gut microbes instead of via mechanoreceptors. One recent advance in the field of WBV research is the increasing awareness that WBV, next to an exercise component, also has a sensory component affecting the brain via skin mechanoreceptors.

**Figure 2.**
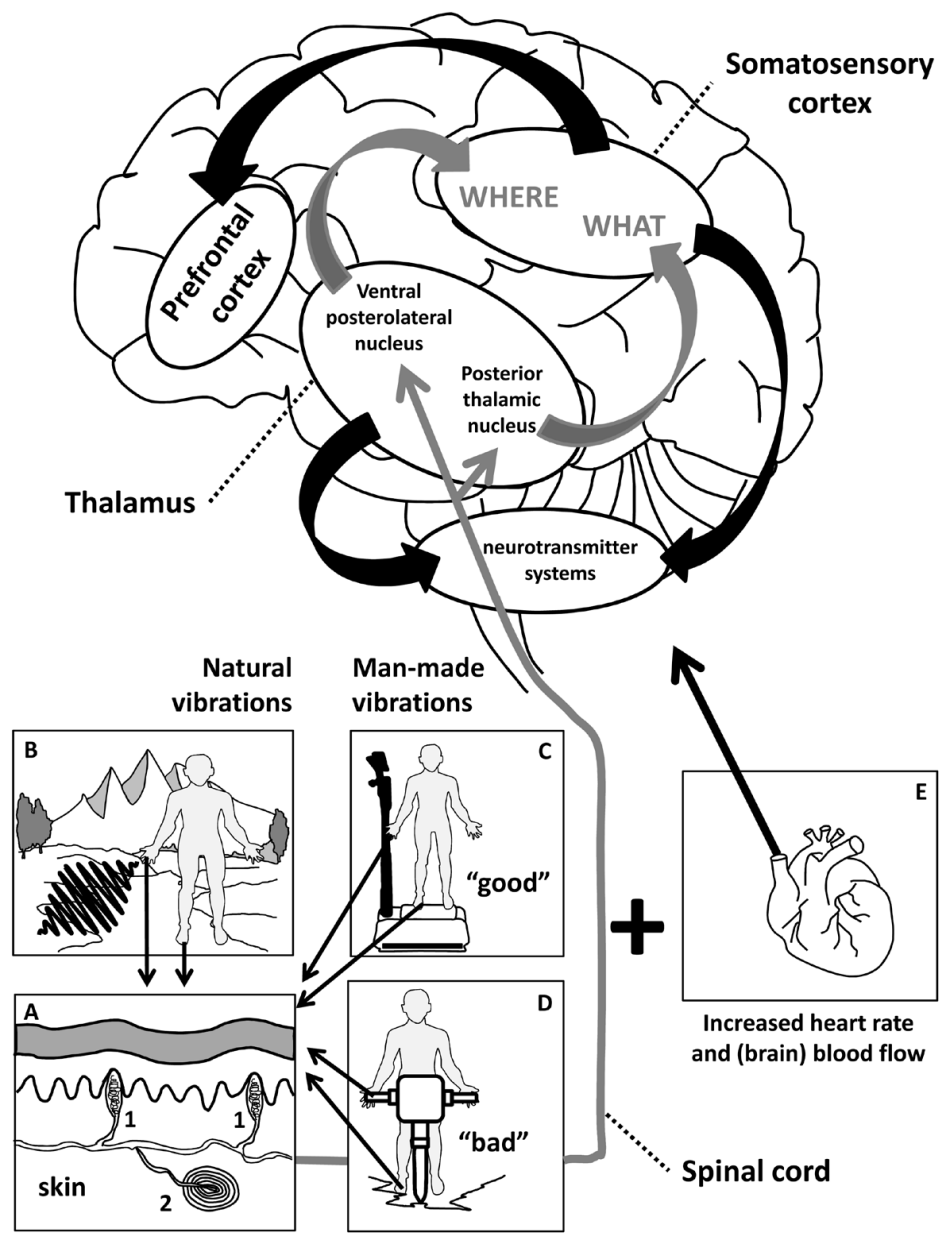
The sensory (skin) component of whole body vibration (WBV). A schematic and simplified overview of vibration detection influencing the brain. Mechanoreceptors in the skin (Panel A: 1. Meissner corpuscles, 2. Pacinian corpuscles) detect the (naturally caused) vibrations (depicted in panel B) and relay the signal to the brain via the spinal cord. In the thalamus, the signal reaches the ventral posterolateral nucleus and the posterior thalamic nucleus. These areas send the signal to the somatosensory cortex, where it has a conscious “where” function (in relation to the body) and a conscious “what” function (in relation to the potential consequences of the detected vibration). The incoming vibrational signal stimulates the prefrontal cortex and neurotransmitter systems, although the pathways of these brain connections are rather unclear. Vibrations can be mimicked by a platform (panel C: potentially “good” vibrations) or by certain (mechanical) tools (panel D: potentially “bad” vibrations). These vibrations have either a positive or a negative impact on human health depending on their features. Detected vibrations can also influence the brain via increased heart rate and hence increased blood flow to the brain.

Humans are endowed with a high density of mechanoreceptors in the skin (notably in the fingertips and feet) to detect vibrations ( Figure 1). Next to those, mechanoreceptors can be found less abundantly in ligament, joints, blood vessels, and organs ^6^. We evolutionarily inherited vibrational sensitivity, which is hard-wired in our body and brain. The mechanoreceptors project via the spinal cord and the thalamus to the somatosensory cortex. Various cortical brain regions are involved in vibrational information processing. Vibrations of high enough energy are therefore consciously detected. Vibrations of low energy or beyond the detection level of hearing (infrasound) still reach the brain, and it is believed that it causes annoyance and distress in many people ^7^. Two main types of cutaneous mechanoreceptors receptive to vibrations are present in mammals, including humans (see 8 for review). Pacinian corpuscles, or pressure receptors, are deeply placed in the skin (and less abundantly elsewhere in the body) ^6^. They sense vibrations at a range of 20–1,000 Hz, with a peak sensitivity around 250 Hz. Meissner corpuscles, or touch receptors, are more superficially placed in the skin. While touching a surface, the skin copies the surface via skin deformations and the corpuscles start to signal with their preferred frequency to the somatosensory cortex (see also Figure 2). Meissner corpuscles sense vibrations at a range of 5–150 Hz, with a peak sensitivity around 10–65 Hz. Meissner and Pacinian corpuscles are mandatory for the detection of vibrations. Stimulation of Meissner corpuscles results in the sensation of tapping-flutter-vibration, whereas stimulation of Pacinian corpuscles results in the sensation of vibration or tickling. Pacinian corpuscles in the human hand also serve a function in active texture exploration ^9^. Under normal conditions, vibrational stimulation of the skin co-activates both types of skin mechanoreceptors either because the range of frequencies of the source is broad or because of harmonics (for example, a 30 Hz vibration will also generate vibrations of 60, 90, 120 Hz etc., although at a lower intensity; see for example ^10^).

## Recent advances in the use of vibrations: medical applications

Humans have a clear ability to detect vibrations of a wide range of frequencies and amplitudes. It had evident evolutionary advantages for individual survival (escaping predators or finding prey, and increased reproduction) for our ancestors. The use of the skin mechanoreceptors for sensing the texture of fruit for its edibility is considered a crucial step in our survival. These original advantages seem not so relevant anymore in our current society. However, vibrations and vibration detection are still linked to health issues. Numerous human studies on vibrations exist and are ongoing, examining under which conditions these vibrations become harmful. The aim of these studies is to determine which regulations are needed to prevent physical or mental damage. In general, vibrations can be dangerous for humans, notably when the amplitude is high (>1 millimeters), the duration is long (> ~30 minutes), and the vibrations have an erratic, random waveform. One aspect of harmful vibrations relates to the principle of resonance. Any part of the body has an intrinsic resonance frequency. The resonance frequencies will induce more physiological effects but if too strong should be considered harmful. For example, it has been estimated that the resonance frequency for the liver is 2–7 Hz in mice, while in humans the resonance frequency for the abdomen is 4–8 Hz, the thorax is 5–10 Hz, and the head is between 20 and 30 Hz ^11^.

WBV research was in part inspired by determining these health risks. In contrast to harmful vibrations or harmful WBV, for example due to tools or driving heavy machinery, WBV can also be beneficial using vibrating platforms (see Figure 1 and Figure 2; “good” versus “bad” vibrations). The mechanical vibration of these vibrating platforms is transferred to the body, which triggers physical and physiological responses (see 12 for review). The type of vibration matters (vertical or side alternating and mechanical or sonic, for example) but also the subject’s posture (sitting or standing on the plate in many different positions). An example of how this can affect the outcome is given by Alizadeh-Meghrazi and co-workers in individuals with spinal cord injuries ^13^. Most often, the waveform is sinusoidal (and hence regular and predictable or predetermined). With a shift in focus from harmful vibrations to beneficial vibrations, the number of WBV publications increased (source: PubMed). Until around 2000, the number of WBV publications was about 15 per year. Then, the yearly number steadily increased and peaked in 2014 and 2015 (about 200 publications per year). The number of publications stabilized around 170 in later years. Systematic reviews started to appear around 2009 and reported on the effects of WBV on a wide variety of topics, including body composition, quality of life, pain management, musculoskeletal morphology and function, pulmonary functions, blood circulation and blood flow, many (neurological) diseases or conditions including musculoskeletal disorders, pulmonary function, chronic obstructive pulmonary disease, diabetes mellitus, fibromyalgia, osteoporosis, cancer, stroke, bone morphology, multiple scleroses, Parkinson’s disease, Alzheimer’s disease, cerebral palsy, and fracture healing. The often-recurrent conclusion of the systematic reviews is that findings are inconsistent, of rather modest effect size if present, and require further research. It signals a field in development with considerable potential but in need of efforts to pinpoint the true value of WBV as a therapy. Is the effect size of WBV interventions large enough to make it clinically relevant for many domains? Increasing awareness of the combined exercise and sensory component in WBV (explained in the next sections) can be viewed as a next step in an attempt to increase the effectiveness of WBV.

## The exercise (muscle) component of WBV

WBV is used in sports as an exercise module. It can improve muscle functioning and bone structure (see 15 for review) in healthy patients as well as those with a medical condition ^16^. Obviously existing WBV exclusion criteria for certain types of patients must be taken into account. WBV-exercise can be a stepping stone for more common forms of physical activity and exercise. Positive findings of WBV as exercise training on numerous outcome parameters keep appearing regularly. Providing a full overview of the recent developments in all of the exercise-related aspects of WBV research is, however, beyond the scope of the current review. In general, several new findings further stress that WBV is potentially a suitable rehabilitation training tool that warrants the continuation of investigations to increase reproducibility and effectiveness (see 17, 18, for example, and references therein). A recent review on WBV as a neuromuscular training method concluded that WBV can bring about improvements in muscle strength, power, and flexibility ^19^. However, it depends not only on the characteristics of the WBV protocol and setting (mentioned above) but also on the characteristics of the participants. However, data are often insufficient to support or refute the use of WBV as an effective therapeutic intervention in diseased conditions. Despite the need for more consistency in WBV research, the steady stream of new publications has brought considerable new insights. Although an object of study since the 60s of the previous century, WBV is now becoming more clearly like a coin with two sides that can be further utilized if it comes to mode of action: the exercise component (e.g. muscles) and the sensory component (e.g. skin).

## The sensory (skin) component of WBV

Obviously, these two components are not mutually exclusive. Skin signals and muscle signals (including proprioceptive signals not further addressed in this review) come together in the associative cortical areas of the brain. Traditionally, the exercise component is associated with WBV-exercise, training, and rehabilitation. However, the existence of the sensory component and the connection to the brain was already recognized in the early days of WBV research (see 12, for example). Focus of the field was still more on the potential discomfort and harmful aspect of WBV on the brain. It was linked to, for instance, motion sickness issues or the impact of vibrations in buildings negatively influencing quality of life ^20^. The dopaminergic brain system becomes activated by WBV (20 Hz, 4G, for 90 minutes ^21^), but by itself this is not necessarily bad for the brain. WBV also increased cerebral blood flow as was shown in a study in which low-frequency WBV protocols were used (ranging from 3–6 Hz) ^22^. A review on peripheral blood flow reported that the change in it is frequency dependent ^23^, and it can be assumed that higher peripheral blood flow also affects the brain. Choi and co-workers showed that 27 Hz WBV (but not 10 Hz WBV) increased brain activity in the prefrontal, motor, and somatosensory cortex ^24^. That WBV can beneficially affect the brain is a rather recent insight. In a series of experiments, it was demonstrated that WBV (30 Hz vibrations) can improve cognitive performance in healthy young and older individuals, including individuals with attention deficits (ADHD) ^25– 29^. An fMRI imaging study in 2016 reported that after a session of WBV (by standing on a platform and providing a non-sinusoidal, random vibration of 7 Hz), the left caudate nucleus showed a significant increase in activity ^30^. A more recent (pilot) study demonstrated the stimulating effects of WBV on the brain by examining electroencephalogram (EEG) activity. Alzheimer’s patients in a relative early stage of the disease received WBV combined with varying standing positions on a platform (20 Hz at the start and every 2 weeks increased by 5 Hz, ending at 40 Hz). EEG activity increased significantly and was accompanied by a significant increase in cognitive performance of the participant ^31^. Our own results with Alzheimer’s patients at a much later stage of the disease (sitting on a WBV platform; see 32 for the experimental design) did not show improvements in cognitive performance. This may suggest that WBV has potential for Alzheimer’s patients at an early stage of the disease only. Obviously, the many differences in the design of the study and the method to apply the vibrations make it difficult to generalize the impact of WBV on the brain. Nevertheless, finding an optimal setting (or settings) in which both components are fully utilized is a future goal in the WBV field.

## The value of translational WBV research

Understanding the underlying (brain) mechanisms of WBV is a crucial step to optimize protocols. Which WBV settings are critical for a given study objective? Translational WBV research (positioning small rodents on a vibrating plate) provides a way to study the underlying mechanisms but only represent ~8% of publications. Of these studies, ~130 were done with rats and ~60 with mice as subjects. Recent advances in the translational approach revealed evidence for both the exercise and the sensory WBV components, but few investigations examined the brain. Ample data are present showing the impact of WBV on targets related to the exercise component of WBV. Providing such an overview is beyond the scope of this review. Only a limited number of (systematic) reviews are available on translational WBV research, but those available are positive about the therapeutic potential of WBV. One older study reviewed WBV data from humans, mice, and rats, and the authors considered WBV as a potentially therapeutic aid with what they called “global ramifications” ^33^. A more recent study reviewed the WBV findings on fracture healing in ovariectomized rats ^34^. Their conclusion, based on nine selected papers, is that WBV can promote bone healing, mineralization, and maturity and restore mechanical properties of bones. What about the sensory (skin) component in relation to the brain? Using cerebral ischemia in rats, researchers found that a 4-week WBV intervention regulated several brain markers in the cortical areas ^35^. Mice subjected to 30 Hz WBV (low amplitude for 5 weeks; 10 minutes per day) showed an improvement in both memory and motor performance ^36^. Brain analyses of mice following this type of WBV treatment revealed a significant increase in the activity of the cholinergic system ^37^. A well-known role of the cholinergic system and its associated receptors is linked to attention ^38, 39^, and enhanced cholinergic activity is thought to contribute to the observed improvements in memory. Changed expression of neurotransmitters in the brain has also been found in an earlier study ^40^ but in the context of long session duration (240 minutes). Long WBV sessions tend to eliminate positive effects on the brain ^36^. A new insight is that the impact of WBV on the brain could also be mediated by the gut–brain axis. The role of the gut microbiome in regulating brain functioning is a booming field of research since the appearance of the seminal paper by Cryan and Dinan in 2012 ^41^. The relevance of a healthy microbiome for general health in humans is now eminent. A recent review provides an overview of the evidence and potential underlying mechanisms ^42^. So far, two papers appeared examining the impact of (sonic) WBV on the microbiome. Song *et al.* ^43^ demonstrated in both humans and mice that WBV affects the gut microbiome in terms of the diversity of the bacterial species present in certain areas of the gut. Yu and co-workers ^44^ demonstrated a gut–microbial link between WBV treatment and the immune system in humans (both in the periphery and in the brain). The functional consequences of these findings cannot be determined easily owing to the complexity of both the gut microbiome and the gut–brain axis. WBV modulating the gut microbiome (possibly directly affecting the microbes) may nevertheless be an issue in future research related to microbial and inflammation-related disorders. In line with this, it was shown that WBV treatment could reduce the level of (neuro)inflammation in a stroke model using rats ^45^.

The use of small rodents to understand human responses to WBV has also several limitations. For one, the rodents stand with four legs on the platform and cannot perform on command additional postures or combine it with exercise. They also possess vibration-sensitive whiskers used for tactile perception ^46^, next to Meissner and Pacinian corpuscles in their paws. But a clear advantage is that they serve mankind as (genetic) models for many human diseases. Unravelling the underlying mechanisms of either beneficial or detrimental WBV effects remains of great value for human disease conditions and well-being.

## Conclusion

It is clear that WBV is a research area that is expanding. It has clear potential for medical applications, but the field could benefit from more standardized and personalized protocols. The use of translational research can facilitate this need. The field should tackle what could be considered the “Big Five” of WBV: vibration amplitude, vibration frequency, method of application, session duration/frequency, and total intervention duration. One step forward will be an update of the guidelines for reporting WBV studies. These new guidelines are currently in progress by a team of WBV experts, preceded by a Delphi study ^47^ in which 56 researchers are involved. In addition, knowledge of the underlying mechanisms should advance further. Once these steps have been made, a much better estimate can be given about the true potential of WBV as a therapeutic intervention and its value as a medical application.
